# Prophylactic Use of Mepitel® Film to Prevent Radiation-Induced Moist Desquamation in Cancer Patients

**DOI:** 10.7759/cureus.42186

**Published:** 2023-07-20

**Authors:** Nahla A Tayyib

**Affiliations:** 1 Faculty of Nursing, Umm Al-Qura University, Makkah, SAU

**Keywords:** wound healing and tissue repair, hydrogel wound dressing, skin lesions, wound complication, pain management, cancer therapy, protective film, radiation injuries, dermatitis, radiotherapy

## Abstract

Cancer patients had limited treatment options for decades, such as surgery, chemotherapy, and radiation therapy, alone or combined. However, there have been substantial improvements in recent years with the introduction of stem cell therapy, hormone therapy, anti-angiogenic treatments, immunotherapy, dendritic cell-based targeted therapy, ablation therapy, nanoparticles, natural antioxidants, radionics, chemodynamic therapy, sonodynamic therapy, and ferroptosis-based therapy. Radiation therapy, or radiotherapy, is a cancer treatment that employs high doses of radiation to eliminate cancer cells and shrink tumors. This treatment is effective as a primary, adjuvant, or palliative therapy. It is an essential, efficient, cost-effective intervention crucial for providing proper palliative oncology care. Although cancer treatment modalities such as intensity-modulated radiotherapy have advanced, they still risk harming the skin and surrounding healthy tissue. Radiotherapy may induce clinical toxicity leading to chronic or acute radiation dermatitis, depending on the toxicity caused by the therapy. Radiation dermatitis, whether in its chronic or acute form, can cause skin shedding that may result in the formation of wounds. Such shedding can also lead to non-healing ulcers and radionecrosis. Mepitel® film helps control radiation-induced moist desquamation in cancer patients. Clinical trials on the prophylactic use of Mepitel film on radiation-induced moist desquamation did not show similarities among patients from various countries; however, the film-based method is more beneficial than other methods. This review examines the various types of dressings utilized in managing radiation-induced dermatitis to enhance wound healing effectiveness while avoiding harm to newly developing tissues. Additionally, this review compares the effectiveness of using Mepitel film for treating radiation-induced moist desquamation to other methods.

## Introduction and background

The basic principle of moist wound healing was one of the essential critical developments in the management of wound dressing. Clinical analysis and experimental data revealed specific required properties for an effective wound dressing. In recent years, advanced technologies were incorporated to show effective dressing that potentially interacts with the wound and improves wound healing. These dressings offer better wound healing processes than traditional methods such as absorbent pads, cotton gauzes, and bandages [1]. Most healthcare workers strongly believe that wound healing cannot be achieved in some cases, and pain and wound trauma are essential considerations for nursing personnel [2,3]. Chronic wound pain is distressing, initiates stress, and further complicates the wound-healing process, which can also lead to depression and the feeling of constant tiredness [4]. Proper removal of wound dressings is crucial in managing wounds in a clinical setting. It helps in the healing process and avoids causing additional pain and trauma [5-7]. When it comes to dressing wounds, traditional materials such as paraffin gauze can sometimes stick to the healing wound and leave behind fabric residue. This can lead to issues such as granuloma or fibrosis, causing further trauma for the patient. These, unfortunately, adhere to healing wounds, and granulation tissue may develop through the fabric of wound dressing. The dried exudates cause trauma and pain in removing epithelial tissue. Recently, the development of novel primary wound contact substances showed a novel approach to managing wound healing [8]. These innovative methods function as a layer between the secondary absorbent material and the wound, providing suitable conditions for wound healing. This material acts as a primary wound contact material. Mepitel® is one of the products developed by Mölnlycke Health Care (Sweden) and is used to dress several types of wounds, such as types of injuries (secondary burns, abrasions, skin tears), skin disorders, chronic wounds, and fixation of the kinds of grafts tissue. Based on the requirements, Mepitel dressing is usually performed for two weeks or more. Acute radiation-induced skin reactions are a significant problem in neck and head cancer and breast cancer patients as well as healthcare professionals [9,10]. In certain cancer cases, patients receive maximum dosage and cause several symptoms associated with acute radiation dermatitis, including moist desquamation, dry desquamation, erythema, and necrosis in some instances [11,12]. The successful use of dressing materials for skin reaction severity has been analyzed with breast cancer patients [13,14] and neck and head cancer patients in some cases. There is still some uncertainty surrounding the mechanism through which Mepitel mitigates skin reactions, especially when it comes to its effectiveness in reducing moist desquamation. Mepitel film has been used as a prophylactic agent in cancer patients, and it prevented moist desquamation and decreased skin reaction severity in >90% of cases in breast cancer patients [9]. Moreover, dressing Mepitel film to neck and head regions is highly complicated and may affect the normal movement of the head and neck and patient compliance. Gillison et al. [15] and Lucas-Roxburgh et al. [16] analyzed the possible use of Mepitel film on moist desquamation in New Zealand and China [9].

In this review, we exclusively analyze the use of film dressing in cancer types. This study critically surveyed the prophylactic application of synthetic Mepitel film used to prevent or control the side effect of radiation-induced moist desquamation in cancer cases. The previous findings were compared with recent innovations in prophylactic biofilm with recent publications. To analyze the current research on the prophylactic application of synthetic Mepitel film, we used keywords such as “Mepitel film,” “prophylactic measures,” “radiation-induced moist desquamation,” “radiation dermatitis,” “cancer and moist desquamation,” “breast cancer and radiation-induced moist desquamation,” and “recent developments in preventive moist desquamation.” These keywords were used to search recent publications in search engines, including PubMed, Web of Science, and Google Scholar. The displayed abstracts and related content were obtained and thoroughly analyzed. We considered articles published in the English language, and other non-English-language publications were not considered. The search was conducted on indexed journal articles and peer-reviewed journals. Letters to the editor, case studies, comments, brief communications, and news were not considered for analysis, and we used only research papers for data processing.

## Review

Wound dressing and management

A wound is considered a complicated problem. Treatment efficiency depends on various aspects, including patient comorbidities, diagnosis, physiological condition, anatomical placement, and wound size. Consequently, the general guideline is that dressing selection is customized to the wound and cancer patient, with the direction and attention of physicians with wound treatment experience [12,13]. This would support patients in receiving the appropriate treatment, achieving maximum efficiency, and avoiding adverse reactions during treatment.

**Figure 1:**
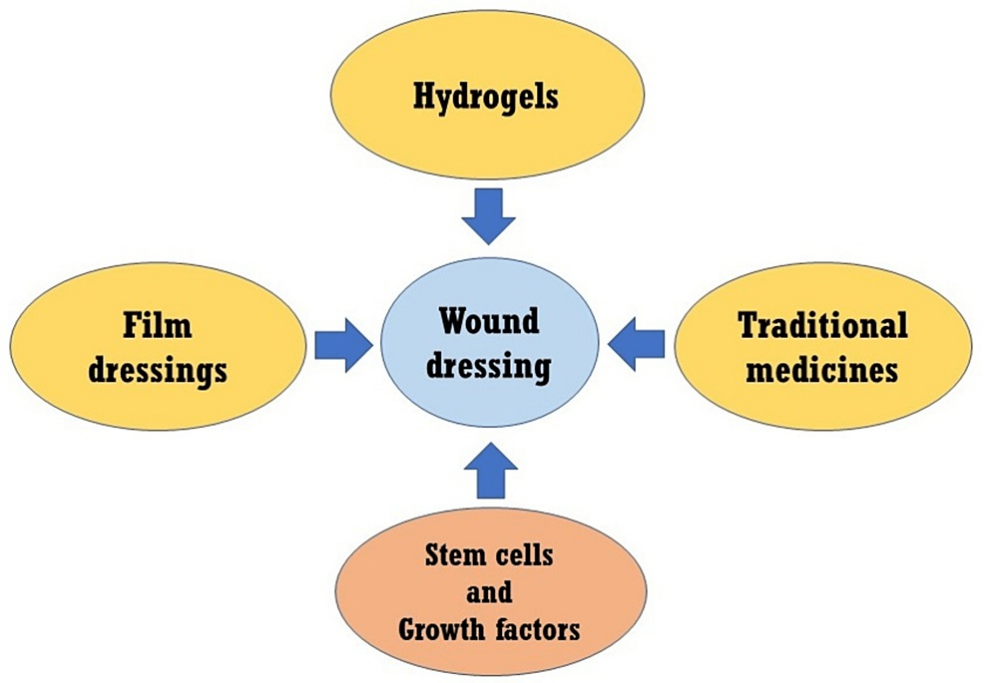
Schematic representation of some dressings utilized in the prevention and treatment of acute radiation dermatitis. There are various choices available, including hydrogels made from biopolymers. Notably, there have been significant advancements in traditional medicine, film dressings, stem cells, and growth factors used for radiation-induced dermatitis.

For radiotherapy-induced changes in the skin, various dressing protocols were established to improve wound management. Non-absorbing materials such as films and absorbing materials such as foams were introduced to control wounds. Introducing substances or other materials alters the skin surface and results in scars, epithelization, and microflora development. Ionizing radiation affects soft tissues for many days to years [17]. Dressings are used in the treatment and prophylaxis of acute radiation dermatitis. These dressings reduce the severity of the dermatitis and pain, improving treatment tolerance. Dressings are made of various materials, including hydrogels, hydrocolloids, alginates, foams, films, membranes, silver-containing materials, and bio-dressing of growth factors and stem cells (Figure 1). Recently introduced wound dressings with outstanding biocompatibility and biodegradability properties are the top choice in wound care.

Hydrogel in wound dressing

Hydrogel dressing is widely used to manage or treat radiotherapy-induced moist desquamation. In a previous study, patients undergoing radiotherapy to the breast, head, or neck region with the development of moist desquamation were selected and treated with hydrogel dressing or 0.5% aqueous gentian violet. The hydrogel dressing significantly improved wound healing in radiotherapy-induced moist desquamation [18]. Hydroactive colloid gel has been used to prevent the development of moist desquamation in breast cancer patients. The hydroactive colloid gel effectively prevented the development of moist desquamation than dexpanthenol application. The hydroactive colloid gel delayed the development of radiotherapy-induced moist desquamation. However, it was statistically not significant (p < 0.01) [19]. The application of calendula cream may help reduce the incidence of Grade 2 or 3 reactions in patients undergoing breast cancer treatment [20]. Sahin et al. [21] used a boron-based gel to prevent the development of moist desquamation in breast cancer cases. Double-blind, parallel-group, experimental studies using human subjects revealed that applying a gel containing 3% sodium pentaborate pentahydrate reduced radioactive impacts and was suggested for clinical applications. Esquirol-Caussa et al. [22] recently performed a pilot study and compared the efficacy of three different creams, namely, HU, HR, and RS, on radiotherapy-induced dermatitis. The HR cream was composed of the active ingredients panthenol-B5, two types of hyaluronic acid, vitamins C and E, and other excipients. The HU cream had the same composition as the HR cream but with a higher fat phase concentration. The RS cream had a composition similar to the HU cream plus the active substance sh-oligopeptide-1 (epidermal growth factor) and vitamin E. The results revealed that the RS cream was more effective in controlling dermatitis during radiotherapy, while the HU cream performed more effectively after the radiotherapy regimen was completed. During radiotherapy, in hypofractionated therapies, the RS cream preserved elasticity and hydration. After radiotherapy, the HR cream further improved elasticity. In summary, these creams considerably controlled the unfavorable effects on the skin. However, their results were not statistically significant because of a low sample size (15 patients, with five patients in each group) [22].

Traditional medicine in the treatment of moist desquamation

Traditional medicines have been used to treat moist desquamation and improve cancer patients’ quality of life. In a study, Erhe Gao cream was prepared using a Chinese medicine formula comprising calamine powder, zinc oxide powder, and lithospermum oil and was carefully applied to the affected skin daily. Traditional medicine effectively prevented the formation of moist desquamation, and the recovery time was 14 days [23]. Nigella sativa L. extract was used to evaluate the activity of avoiding the chance of acute radiation dermatitis in cancer cases. The applied gel delayed the onset of moist desquamation and acted as a pain-relieving material in breast cancer patients [24]. A flavonoid compound, silymarin, was extracted from *Silybum marianum*, and its role in preventing radiodermatitis in breast cancer patients was tested. A total of 40 patients undergoing radiotherapy were selected, and the gel was applied for five weeks continuously. Applying silymarin gel delayed and decreased the severity of radiodermatitis [25]. The nano-curcumin capsules were used to study the impact on radiotherapy-mediated skin damage. When compared to the control group, simultaneous administration of the nano-curcumin supplement did not substantially lower radiation-induced skin reaction severity (p > 0.05); however, there was a significant difference at week seven (p = 0.01). Moreover, patient-reported pain was considerably lower in the treatment group compared to the control group. Administering nano-curcumin possibly reduced radiation-induced skin damage in breast cancer patients. However, this benefit was not statistically significant [26].

The processes of radiosensitization and radioprotection mediated by soy isoflavones have been addressed, emphasizing the function of soy isoflavones in boosting radiation impact on tumors and decreasing radiation-induced inflammatory responses in normal tissues. Soy isoflavones might be employed as a safe, nontoxic supplementary technique to increase radiation efficacy on cancer while limiting harm to normal tissues in the radiation field [27]. The composition of bioactive secondary metabolites of brown seaweed, such as phlorotannins (PTNs), have anti-inflammatory activities, but their therapeutic value is limited. Previously, Yang et al. investigated the effect of PTNs in vivo in a mouse model of radiation dermatitis. Compared to the vehicle control, the macroscopic radiation dermatitis score showed that PTNs significantly reduced radiation dermatitis. Histopathological studies of skin tissues revealed that PTNs reduced epidermal and dermal thickness [28]. Various dressing materials were analyzed to test their compatibility in acute radiation dermatitis prophylaxis and treatment. The principal objective of the application was the complete reduction of acute radiation dermatitis severity-related symptoms, such as pain and discomfort. The development of moist desquamation could not be entirely prevented by radiotherapy. A small, qualitative study reported several benefits to using polymeric membrane dressings to manage patients presenting with radiotherapy-induced skin damage [29].

Radiotherapy-induced dermatitis in cancer patients

**Figure 2:**
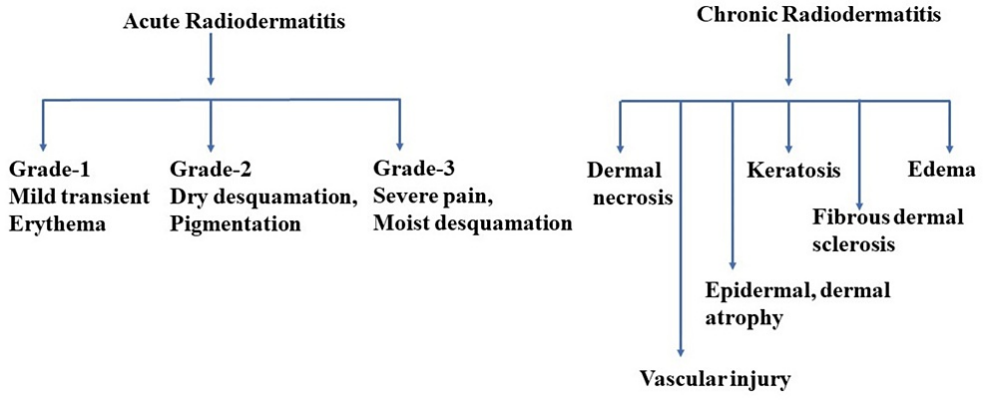
Schematic representation of clinical manifestations and symptoms of acute and chronic radiodermatitis. Acute radiodermatitis is defined by skin lesions that appear within 90 days of radiotherapy. During the first week of radiotherapy, acute radiodermatitis (Grade 1) may cause mild and transient redness on the skin (erythema). By the second week, dryness and peeling (desquamation) may occur due to dehydration in Grade 2. Within three to six weeks, severe pain and moist peeling (desquamation) may develop in Grade 3 dermatitis. Chronic radiodermatitis develops more than 90 days after the completion of radiation treatment. In some cases, it may occur after six months or years after irradiation. Symptoms such as dermal necrosis, keratoses, edema, fibrosis, dermal sclerosis, pigmentation, epidermal or dermal atrophy, vascular injury, and telangiectasia are common. Modified from Iacovelli et al. [33].

Radiotherapy is a well-established cancer treatment that can effectively target and treat various types of cancers, including breast cancer [30]. Radiotherapy-induced dermatitis in cancer patients is a common side effect in almost all cancer patients undergoing treatment (Figure 2). The quality of the radiation beam also influences the development of acute skin toxicity. The total radiation dose, dose/fraction, beam characteristics, volume, and surface area of radiation exposure influence the degree of tissue damage. Advanced radiotherapy modalities such as intensity-modulated radiotherapy reduce radiation injury of the skin by delivering more homogenous radiation than traditional wedge beam radiation [31,32]. Skin is one of the major organs that receive high doses of radiation. This is considered a radiosensitive organ, and severe damage has been reported due to the high doses of radiation. Moist desquamation is one of the major types, and it affects the lifestyle of patients and causes discomfort in cancer cases. The development of patchy moist desquamation is considered Grade II acute radiation dermatitis. Moist desquamation other than creases and skin folds, abrasion, and minor trauma-induced bleeding is referred to as Grade III type [10,11,33].

Characteristics of film used in wound dressing

**Figure 3:**
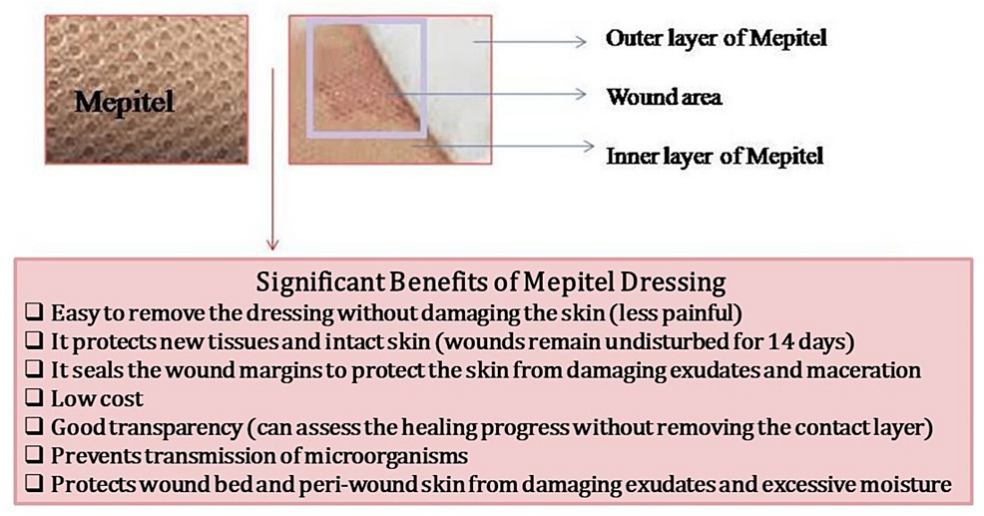
Major benefits of Mepitel film dressing in cancer patients.

Films are thin, transparent polyurethane materials and are flexible, designed to adhere to the surrounding and wound skin and manage moisture content in the affected places. They are formed using semi-permeable materials because of their increased permeability to oxygen, water vapor, and carbon dioxide. The film is not permeable to microorganisms and water. It has solid adhesive properties and helps dress in moving areas such as joints. The film material was prepared using nylon and was not commonly recommended because of its limited absorption potential. The film is one of the welcoming materials because of its transparent properties and elastic nature, and it is beneficial for testing wound closure without removing dressing materials. Film dressing helps cover superficial and newly healed wounds, including split-skin graft regions and intravenous catheter sites (Figure 3). One of the problems of using the film is preventing fluid accumulation at the wound site. The generated exudates can affect the adhesive properties of the film and the chance to break the dressing and threaten the growth of microorganisms [13,34].

Recently, biomaterials have been employed to protect injured tissues from moist desquamation. This method used traditional fibers with bioactive substances such as stem cells or growth factors to improve the healing process. These materials may help treat severe acute radiation dermatitis. Foam dressing coated with epidermal growth factor improved the healing process in neck and head cancer patients [35]. Polymeric membranes are highly useful than other methods to treat acute radiation dermatitis, which was tested by Hegarty and Wong [36]. Hydrofilm (polyurethane film) is another polymeric dressing applied in acute radiation dermatitis prophylaxis. The designed film reduced the chronic effect of radiation [14]. However, experimental trials revealed good film protection against moist desquamation [37]. These earlier clinical studies showed complete abatement of dermatitis symptoms and 100% prevention of moist desquamation. Moreover, in the study group, symptoms such as burning, itching, and pain were not observed. Soft silicone film has been used as the protective material and has been confirmed by randomized controlled trials [38]. The hydrocolloid and hydrogel dressings were applied in treating severe and moderate acute radiation dermatitis. The selected material reduced the development of moist desquamation and maintained a wet condition at the injury site. As an alternative to the film dressing, hydrogel has been used in several trials to analyze its efficacy for treating moist desquamation in neck, head, and breast cancer patients. The use of hydrogels has shown improved protective properties and healing rates in patients affected by moist desquamation [18]. Silver-leaf nylon dressings were used to treat breast cancer patients. The silver-leaf nylon dressing did not show significant results in the later stages [39].

Mepitel and its advantage in tissue repair

Mepitel films are transparent polyurethane, thin, and flexible materials designed for the wound and to manage moisture content in the affected places. This film increases permeability to oxygen, water vapor, and carbon dioxide [40,41]. Mepitel film is a semi-permeable material prepared based on Safetac technology. It is a semi-transparent, highly porous film with a very low-adherent wound contact layer, consisting of a polyamide net (flexible) with very soft silicone. Generally, the silicone coating is moderately tacky, which involves the retention of the dressing to the peri-wound area. The adhesion procedure effectively controls maceration by affecting the lateral movement of exudates from the wound. The application of Mepitel reduces pain and protects new tissue without any damage. Naturally, Mepitel is not an absorbent material but contains pores or apertures about 1 mm in diameter that allow the movement of exudates into a second layer of absorbent dressing. It is used in wound management, mainly skin abrasions, tears, second-degree burns, surgical excisions, lacerations, epidermolysis bullosa, and skin damage following steroid therapy or radiotherapy. Mepitel is sterilized by ethylene oxide and is available in various sizes (2 cm × 7 cm to 20 cm × 32 cm).

Mepitel film is helpful to use as a prophylactic material and is beneficial to dress the wound from the first day of radiotherapy. The primary significant advantage of film dressing is transparency and monitoring the development of wound healing. Foam dressing comprises an absorbent layer (polyurethane film) and a silicone layer (close contact with the wound). Nanocrystalline silver-coated material is used for the preparation of silver nylon dressing material. These silver-coated materials are effective against Gram-negative and Gram-positive bacteria as well as against various fungal pathogens. Advanced dressings made from stem cells, growth factors, and fibers are now available to treat acute radiation injuries. These innovative materials have been carefully developed and are proving to be highly effective [42]. Calcium alginate materials and non-adherent absorbent dressing are also used to treat moist desquamation [5,8,9]. The application of Mepitel film reduced the development of moist desquamation, and the continuous use reduced pain and provided better relief [43]. Recently, a Mepitel film was used as a non-adherent wound dressing for radiation dermatitis in neck and head cancer patients.

Morgan [12] described the use of Mepitel film in the management of breast cancer cases. Mepitel film is easy to handle by healthcare professionals, potentially reducing adverse side effects on skin tissues and improving patient health. Mepitel film dressing has several advantages over foam dressing and is helpful in the case of moist desquamation. Moreover, no gold standard guidelines have been developed to treat radiation-induced skin injury [13]. Hydrofilm is recommended to manage desquamation and reduce pain and itching at the site of radiation-induced tissue injury [14]. Mepilex Lite dressings have been used to care for nasopharyngeal carcinoma patients to heal post-irradiation dermatitis. Mepilex Lite dressings improved wound healing, decreased healing time and pain, and improved neck movement and mental health. The median healing time was 23 days in the control patients and only 16 days in the experimental groups treated with Mepilex Lite dressings [34]. Among the dressing materials and methods, film dressing is considered one of the superior methods for skin management.

Film dressing in head and neck cancer

A silicone-based film-forming gel dressing (StrataXRT®) was used to manage head and neck cancer patients in a randomized control trial. The used film, StrataXRT®, effectively prevented and delayed the development of skin toxicity in cancer patients with radiation dermatitis [44]. Mepitel film was used in squamous cell carcinoma of head and neck cancer patients, and it was found that these films were unsatisfactory in the selected cases [45]. Wooding et al. [46] used Mepitel film to reduce radiation-induced skin reactions in neck and head cancer patients in clinical trials. Their findings revealed that Mepitel film reduced skin reactions in patients from New Zealand and China. Mepitel film and Biafine cream were compared to analyze the efficacy of head and neck cancer patients subjected to radiation. The patients were subjected to a dose of 50 Gy in 25 fractions in the neck region, and the right and left lymph nodes were subjected to Biafine cream or Mepitel film as a prophylactic measure. The results revealed an excellent protective role of Mepitel film over Biafine cream and reduced the incidence of moist desquamation in neck and head cancer patient cohorts. Mepitel film has been recommended in various cases because it is semi-permeable, and the dressing is based on Safetac technology. The film can be used continuously from day one of radiotherapy to the final day without changing the material. Due to its transparency, it is widely recommended [47].

Film dressing in breast cancer

The prophylactic use of Mepitel film on moist desquamation was analyzed in breast cancer patients. The patients receiving radiation were randomized to either aqueous cream or Mepitel film, and the skin severity was assessed. The disease severity was reduced by skin reaction severity (>92%) in patients randomized with Mepitel film, and no development of moist desquamation was observed [48] in prophylactic treatment. The prophylactic use of Mepitel film in radiation dermatitis was tested by Wan et al. [49]. Breast cancer patients treated with Mepitel had a reduced incidence of radiation dermatitis. Moreover, the lack of randomized experimental trials delayed the use of Mepitel as a globally recognized prophylactic substance. Yee et al. [50] used Mepitel film to analyze its preventive role in breast cancer patients suffering from radiation dermatitis in Canada. They found that none of the enrolled patients developed Grade 3 radiation dermatitis, 17.9% developed Grade 2 radiation dermatitis, 10.7% developed Grade 2 radiation dermatitis, 10.7% developed moist desquamation, and brisk erythema was reported in 7.1% of patients. This study did not reveal complete prevention of moist desquamation in breast cancer patients. Mepitel film reduced the pain and sensitivity of Danish breast cancer patients exposed to radiotherapy. The film-covered area showed good relief, and patients preferred to cover the entire treatment area to reduce pain [51]. The latest study by Fuerst [52] reported the use of Mepitel film in breast cancer patients to improve their health. The benefits of the film include reducing the development of Frade 3 radiation dermatitis and moist desquamation; only 8% of cases showed symptoms of moist desquamation. Mepitel film is widely used to prevent moist desquamation in breast cancer patients. The application of Mepitel film reduced moist desquamation and skin symptoms [53]. Moreover, using Mepitel film completely prevented moist desquamation in one randomized experimental trial, and other randomized trials did not reveal 100% protection against moist desquamation (Table 1).

**Table 1:** Clinical trials demonstrating the role of Mepitel film in preventing radiation-induced moist desquamation in cancer patients.

Reference	Sample size (n)	Cancer type	Dressing material	Result
Schmeel et al. [9]	62	Breast cancer	Polyurethane film	100% prevention of moist desquamation
Herst et al. [35]	78	Breast cancer	Mepitel film	>92% reduction of moist desquamation
Wan et al. [36]	78	Head and neck cancer	Mepitel film	27% reduction of moist desquamation
Wooding et al. [33]	36	Breast cancer	Mepitel film	29% reduction of radiation-induced skin reaction
Yan et al. [40]	44	Breast cancer	Mepitel film	41% reduction of moist desquamation
Yee et al. [37]	30	Breast cancer	Mepitel film	>89% reduction of moist desquamation
Moller et al. [38]	101	Breast cancer	Mepitel film	82% reduction of skin damage

## Conclusions

Wound dressing is an excellent strategy for wound care and therapy, as evidenced by the favorable findings of various trials. However, further research and development are needed to validate modern dressing methods. Currently available dressing methods are critical to establishing a moist environment for wound healing, pain relief, and antibacterial protection. Unfortunately, only a few clinical trials have been conducted to assess the efficacy of these popular modern dressings. Trials on wound dressing effectiveness are typically performed on less complicated wounds, resulting in oversimplification of the actual clinical aspects.

Further clinical studies and research are required to establish the safety and efficacy of several types of latest wound dressing methods. Analyzing novel materials for modern wound dressings with positive outcomes exhibited via numerous tests has resulted in innovation in enhancing the dressing materials available in the market. The recent trend in developing ideal dressing products has several advantages. It has various health benefits, including increased antibacterial activities, environmental benefits, high biological compatibility, eco-friendly, and ease of handling, especially with natural polymers. New materials have been designed to respond to the various situations and stages of wound healing.

This literature review discussed the use of Mepitel film in acute radiation dermatitis prophylaxis. Among the available methods, foam dressing and Mepitel film dressing are helpful. Moreover, Mepitel film showed >90% success among patients worldwide. The use of Mepitel film is cost-effective and prevents radiation-induced moist desquamation. Based on the available publications and supporting evidence, Mepitel film is a beneficial aid for wound healing and can prevent radiation-induced Grade 2 or 3 dermatitis. As a result, it is highly recommended for individuals undergoing treatment for neck, head, or breast cancer.

## References

[1] Thomas S (2022). The role of dressings in the treatment of moisture-related skin damage. http://www.worldwidewounds.com/2008/march/Thomas/Maceration-and-the-role-of-dressings.html.

[2] Frykberg RG, Banks J (2015). Challenges in the treatment of chronic wounds. Adv Wound Care (New Rochelle).

[3] Heerschap C, Nicholas A, Whitehead M (2019). Wound management: Investigating the interprofessional decision-making process. Int Wound J.

[4] Price P, Fogh K, Glynn C, Krasner DL, Osterbrink J, Sibbald RG (2007). Managing painful chronic wounds: the Wound Pain Management Model. Int Wound J.

[5] Price PE, Fagervik-Morton H, Mudge EJ (2008). Dressing-related pain in patients with chronic wounds: an international patient perspective. Int Wound J.

[6] Brölmann FE, Vermeulen H, Go P, Ubbink D (2013). [Guideline 'Wound Care': recommendations for 5 challenging areas]. Ned Tijdschr Geneeskd.

[7] Dhivya S, Padma VV, Santhini E (2015). Wound dressings - a review. Biomedicine (Taipei).

[8] Okur ME, Karantas ID, Şenyiğit Z, Üstündağ Okur N, Siafaka PI (2020). Recent trends on wound management: new therapeutic choices based on polymeric carriers. Asian J Pharm Sci.

[9] Paterson D (2012). Randomized intra-patient controlled trial of Mepilex Lite dressings versus aqueous cream in managing radiation-induced skin reactions postmastectomy. J Cancer Sci Ther.

[10] Palma G, Monti S, Conson M (2020). NTCP models for severe radiation induced dermatitis after IMRT or proton therapy for thoracic cancer patients. Front Oncol.

[11] Chen MF, Chen WC, Lai CH, Hung CH, Liu KC, Cheng YH (2010). Predictive factors of radiation-induced skin toxicity in breast cancer patients. BMC Cancer.

[12] Morgan K (2014). Radiotherapy-induced skin reactions: prevention and cure. Br J Nurs.

[13] Yang X, Ren H, Guo X, Hu C, Fu J (2020). Radiation-induced skin injury: pathogenesis, treatment, and management. Aging (Albany NY).

[14] Schmeel LC, Koch D, Stumpf S (2018). Prophylactically applied Hydrofilm polyurethane film dressings reduce radiation dermatitis in adjuvant radiation therapy of breast cancer patients. Acta Oncol.

[15] Gillison ML, Chaturvedi AK, Anderson WF, Fakhry C (2015). Epidemiology of human papillomavirus-positive head and neck squamous cell carcinoma. J Clin Oncol.

[16] Lucas-Roxburgh R, Benschop J, Dunowska M, Perrott M (2015). Prevalence of human papillomaviruses in the mouths of New Zealand women. N Z Med J.

[17] (2023). Ionizing radiation, health effects and protective measures. https://www.who.int/news-room/fact-sheets/detail/ionizing-radiation-health-effects-and-protective-measures.

[18] Gollins S, Gaffney C, Slade S, Swindell R (2008). RCT on gentian violet versus a hydrogel dressing for radiotherapy-induced moist skin desquamation. J Wound Care.

[19] Censabella S, Claes S, Orlandini M, Braekers R, Bulens P (2017). Efficacy of a hydroactive colloid gel versus historical controls for the prevention of radiotherapy-induced moist desquamation in breast cancer patients. Eur J Oncol Nurs.

[20] McQuestion M (2011). Evidence-based skin care management in radiation therapy: clinical update. Semin Oncol Nurs.

[21] Sahin F, Pirouzpanah MB, Bijanpour H (2022). The preventive effects of boron-based gel on radiation dermatitis in patients being treated for breast cancer: a phase III randomized, double-blind, placebo-controlled clinical trial. Oncol Res Treat.

[22] Esquirol Caussa J, Ribes Bernal JL (2023). Clinical assessment of specifically formulated creams for oncology: case series. Preprints.org.

[23] Xiaoshan W, Zhixi L, Liang L, Shuchun L, Xia W, Yuyi W, Feng L (2014). Treating breast cancer radiotherapy-induced moist desquamation with a traditional Chinese medicine formula: a case series pilot study. J Altern Complement Med.

[24] Rafati M, Ghasemi A, Saeedi M, Habibi E, Salehifar E, Mosazadeh M, Maham M (2019). Nigella sativa L. for prevention of acute radiation dermatitis in breast cancer: a randomized, double-blind, placebo-controlled, clinical trial. Complement Ther Med.

[25] Karbasforooshan H, Hosseini S, Elyasi S, Fani Pakdel A, Karimi G (2019). Topical silymarin administration for prevention of acute radiodermatitis in breast cancer patients: a randomized, double-blind, placebo-controlled clinical trial. Phytother Res.

[26] Talakesh T, Tabatabaee N, Atoof F, Aliasgharzadeh A, Sarvizade M, Farhood B, Najafi M (2022). Effect of nano-curcumin on radiotherapy-induced skin reaction in breast cancer patients: a randomized, triple-blind, placebo-controlled trial. Curr Radiopharm.

[27] Hillman GG (2019). Soy isoflavones protect normal tissues while enhancing radiation responses. Semin Radiat Oncol.

[28] Yang K, Kim SY, Park JH (2020). Topical application of phlorotannins from brown seaweed mitigates radiation dermatitis in a mouse model. Mar Drugs.

[29] Scott A (2014). Polymeric membrane dressings for radiotherapy-induced skin damage. Br J Nurs.

[30] Baskar R, Lee KA, Yeo R, Yeoh KW (2012). Cancer and radiation therapy: current advances and future directions. Int J Med Sci.

[31] Hall E, Cox J (2003). Physical and biological basis of radiation therapy. Radiation Oncology.

[32] Pignol JP, Olivotto I, Rakovitch E (2008). A multicenter randomized trial of breast intensity-modulated radiation therapy to reduce acute radiation dermatitis. J Clin Oncol.

[33] Iacovelli NA, Torrente Y, Ciuffreda A, Guardamagna VA, Gentili M, Giacomelli L, Sacerdote P (2020). Topical treatment of radiation-induced dermatitis: current issues and potential solutions. Drugs Context.

[34] Zhong WH, Tang QF, Hu LY, Feng HX (2013). Mepilex Lite dressings for managing acute radiation dermatitis in nasopharyngeal carcinoma patients: a systematic controlled clinical trial. Med Oncol.

[35] Lee J, Lee SW, Hong JP, Shon MW, Ryu SH, Ahn SD (2016). Foam dressing with epidermal growth factor for severe radiation dermatitis in head and neck cancer patients. Int Wound J.

[36] Hegarty F, Wong M (2014). Polymeric membrane dressing for radiotherapy-induced skin reactions. Br J Nurs.

[37] Burke G, Faithfull S, Probst H (2022). Radiation induced skin reactions during and following radiotherapy: a systematic review of interventions. Radiography (Lond).

[38] Zou MY, Xu DJ, Zhang R (2021). Study on prevention of acute radiodermatitis with soft silicone film dressing. Indian J Pharm Sci.

[39] Aquino-Parsons C, Lomas S, Smith K, Hayes J, Lew S, Bates AT, Macdonald AG (2010). Phase III study of silver leaf nylon dressing vs standard care for reduction of inframammary moist desquamation in patients undergoing adjuvant whole breast radiation therapy. J Med Imaging Radiat Sci.

[40] (2023). Mepitel film with Safetac Technology - Gentry Health. https://gentryhealth.co.za/product/mepitel-film-with-safetac-technology/.

[41] Bugmann P, Taylor S, Gyger D (1998). A silicone-coated nylon dressing reduces healing time in burned paediatric patients in comparison with standard sulfadiazine treatment: a prospective randomized trial. Burns.

[42] Kolimi P, Narala S, Nyavanandi D, Youssef AA, Dudhipala N (2022). Innovative treatment strategies to accelerate wound healing: trajectory and recent advancements. Cells.

[43] Oshin F, McBrayne L, Bratt M (2020). A retrospective chart review on the prophylactic use of Mepitel film for breast cancer patients undergoing chest wall irradiation: a single-institution experience. J Med Imaging Radiat Sci.

[44] Chan RJ, Blades R, Jones L (2019). A single-blind, randomised controlled trial of StrataXRT® - a silicone-based film-forming gel dressing for prophylaxis and management of radiation dermatitis in patients with head and neck cancer. Radiother Oncol.

[45] Rades D, Narvaez CA, Splettstößer L (2019). A randomized trial (RAREST-01) comparing Mepitel® film and standard care for prevention of radiation dermatitis in patients irradiated for locally advanced squamous cell carcinoma of the head-and-neck (SCCHN). Radiother Oncol.

[46] Wooding H, Yan J, Yuan L, Chyou TY, Gao S, Ward I, Herst PM (2018). The effect of Mepitel film on acute radiation-induced skin reactions in head and neck cancer patients: a feasibility study. Br J Radiol.

[47] Fernández-Castro M, Martín-Gil B, Peña-García I, López-Vallecillo M, García-Puig ME (2017). Effectiveness of semi-permeable dressings to treat radiation-induced skin reactions. A systematic review. Eur J Cancer Care (Engl).

[48] Herst PM, Bennett NC, Sutherland AE, Peszynski RI, Paterson DB, Jasperse ML (2014). Prophylactic use of Mepitel film prevents radiation-induced moist desquamation in an intra-patient randomised controlled clinical trial of 78 breast cancer patients. Radiother Oncol.

[49] Wan BA, Chan S, Herst P (2019). Mepitel film and Mepilex lite for the prophylaxis and treatment of skin toxicities from breast radiation. Breast.

[50] Yee C, Lam E, Gallant F (2021). A feasibility study of Mepitel film for the prevention of breast radiation dermatitis in a Canadian center. Pract Radiat Oncol.

[51] Møller PK, Olling K, Berg M (2018). Breast cancer patients report reduced sensitivity and pain using a barrier film during radiotherapy - a Danish intra-patient randomized multicentre study. Tech Innov Patient Support Radiat Oncol.

[52] Fuerst ML (2023). Film dressing reduces acute radiation dermatitis in breast cancer. Oncol Times.

[53] Yan J, Yuan L, Wang J, Li S, Yao M, Wang K, Herst PM (2020). Mepitel film is superior to Biafine cream in managing acute radiation-induced skin reactions in head and neck cancer patients: a randomised intra-patient controlled clinical trial. J Med Radiat Sci.

