# Correction: Prophylactic Use of Mepitel® Film to Prevent Radiation-Induced Moist Desquamation in Cancer Patients

**DOI:** 10.7759/cureus.c131

**Published:** 2023-08-14

**Authors:** Nahla A Tayyib

**Affiliations:** 1 Faculty of Nursing, Umm Al-Qura University, Makkah, SAU

This article has been corrected due to multiple inaccurate statements that are described in detail below. The journal greatly regrets that these errors were not identified by the journal editorial staff or external peer reviewers prior to publication.

The article states, "Recently, a Mepitel film was used as a polymeric membrane dressing for radiation dermatitis in neck and head cancer patients." However, Mepitel is not a polymeric membrane dressing. This statement has been corrected to read as follows: "Recently, a Mepitel film was used as a non-adherent wound dressing for radiation dermatitis in neck and head cancer patients."

In addition, https://www.magonlinelibrary.com/doi/abs/10.12968/bjon.2014.23.Sup10.S24" the study by Audrey Scott that is cited as reference #29 is misrepresented in both study type and results. In reference to Scott's article, it is stated that, "A randomized experimental trial reported inconclusive results on using polymeric membrane dressings to manage cancer patients [29]." The study in question was a qualitative study, not a randomized experimental trial, and the results were not inconclusive. Scott found that PolyMem dramatically reduced pain and inflammation (the topic of the paragraph). "...when comparing the notes with the patient free-text diaries, the information gathered supported the conclusion that the dressing had an impact on both a reduction in pain and healing rates. Patients reported a specific reduction in wound pain and increased comfort when using the dressing."

As a result, the above statement has been amended to read as follows: "A small, qualitative study reported several benefits to using polymeric membrane dressings to manage patients presenting with radiotherapy-induced skin damage [29]."

